# Why high cholesterol levels help hematological malignancies: role of nuclear lipid microdomains

**DOI:** 10.1186/s12944-015-0175-2

**Published:** 2016-01-12

**Authors:** Michela Codini, Samuela Cataldi, Andrea Lazzarini, Anna Tasegian, Maria Rachele Ceccarini, Alessandro Floridi, Remo Lazzarini, Francesco Saverio Ambesi-Impiombato, Francesco Curcio, Tommaso Beccari, Elisabetta Albi

**Affiliations:** Department of Pharmaceutical Sciences, University of Perugia, Perugia, Italy; Laboratory of Nuclear Lipid BioPathology, CRABiON, Perugia, Italy; Department of Clinical and Biological Sciences, University of Udine, Udine, Italy

**Keywords:** β-actin, Hypercholesterolemia, Lymphoma cells, Sphingomyelin, Sphingomyelinase

## Abstract

**Background:**

Diet and obesity are recognized in the scientific literature as important risk factors for cancer development and progression. Hypercholesterolemia facilitates lymphoma lymphoblastic cell growth and in time turns in hypocholesterolemia that is a sign of tumour progression. The present study examined how and where the cholesterol acts in cancer cells when you reproduce in vitro an in vivo hypercholesterolemia condition*.*

**Methods:**

We used non-Hodgkin’s T cell human lymphoblastic lymphoma (SUP-T1 cell line) and we studied cell morphology, aggressiveness, gene expression for antioxidant proteins, polynucleotide kinase/phosphatase and actin, cholesterol and sphingomyelin content and finally sphingomyelinase activity in whole cells, nuclei and nuclear lipid microdomains.

**Results:**

We found that cholesterol changes cancer cell morphology with the appearance of protrusions together to the down expression of β-actin gene and reduction of β-actin protein. The lipid influences SUP-T1 cell aggressiveness since stimulates DNA and RNA synthesis for cell proliferation and increases raf1 and E-cadherin, molecules involved in invasion and migration of cancer cells. Cholesterol does not change GRX2 expression but it overexpresses SOD1, SOD2, CCS, PRDX1, GSR, GSS, CAT and PNKP. We suggest that cholesterol reaches the nucleus and increases the nuclear lipid microdomains known to act as platform for chromatin anchoring and gene expression.

**Conclusion:**

The results imply that, in hypercholesterolemia conditions, cholesterol reaches the nuclear lipid microdomains where activates gene expression coding for antioxidant proteins. We propose the cholesterolemia as useful parameter to monitor in patients with cancer.

## Background

Cholesterol (CHO)-rich diet is now considered an important risk factor for cancer development [1–3]. However, although detrimental effect of high level of CHO for cancer progression have been increasingly noted, we do not know the mechanisms involved. Llaverias et al. [4] reported that hypercholesterolemia (hyperCHO), in association with oncogenic stimuli, leaded to accelerated tumour formation with subsequent hypocholesterolemia (hypoCHO). Already Iso et al. [5] had previously shown that patients with liver cancer often exhibited hypoCHO, which seemed to be a symptom rather than a cause of cancer. We demonstrated that severe hypoCHO was a sign of cancer progression in patients with acute lymphoid leukemia [6]. In vitro, when serum CHO concentration was 800 nM corresponding to 280 mg/dl in the blood of patients, non-Hodgkin’s T cell human lymphoblastic lymphoma cells (SUP-T1) incorporated CHO with avidity and used it for their proliferation [6]. Therefore normal CHO concentration did not influence tumour growth; otherwise hyperCHO facilitated the entry of CHO into the cells by stimulating cell growth and inducing severe hypoCHO [6]. Recently it has been highlighted that cell membrane specific lipid microdomains (LMs) rich in CHO and sphingomyelin (SM) or lipid rafts controlled cancer cell signalling [7, 8]. Cancer progression involved dysregulation of lipid rafts [9]. Increased CHO content in lipid rafts is associated with greater survival of prostate cancer cells [10]. After cell treatment with CHO disrupting or usurping agents, raft-associated proteins and lipids were dissociated and disease severity was mitigated [11].

We previously demonstrated the presence of LMs in the nucleus, in association with inner nuclear membrane (Nuclear Lipid Microdomains, NLMs), where they acted as platform for active chromatin anchoring, DNA duplication and transcription process [12, 13]. CHO-SM relationship in NLM was different in normal and cancer cells [14]. Dexamethasone localized in NLM where it acted as inhibitor of SUP-T1 cell growth [15].

Plasma membrane was supported by an actin network. The dynamic regulation of actin polymerization into filaments and depolymerization into monomers was the key for its many functions during cell motility, intracellular transport and cell shape maintenance [16]. Regulation of the actin cytoskeleton was imperative to normal cell function. Tumour invasion and metastasis were increasingly being associated with deregulation of the actin system [17]. In the diffuse large B-cell lymphoma a β-actin mutation was identified [18].

Despite the anticancer role of antioxidant enzymes has been widely demonstrated, proliferation, invasiveness, migration, apoptosis and drug resistance of cancer have been associated with the over-expression of antioxidant enzymes [19]. Superoxide dismutases (SODs) are a family of enzymes responsible for the detoxification of ROS; copper-zinc SOD1 is the predominant dismutase in the cytoplasm whereas manganese SOD2 is located in the mitocondria and SOD3 is extracellular [20]. The association with the copper chaperone for SOD (CCS) delivers Cu to SOD1, forms disulfide bond and induces activation of copper/zinc SOD1 [21]. Glutathione (GSH) is one of the main antioxidants. The oxidized form of GSH (GSSG) is reduced back to GSH by the NADPH-dependent catalysis of the flavoenzyme GSH reductase (GSR); *the novo* GSH biosynthesis requires glutathione synthase (GSS) enzyme [22]. Peroxiredoxins (PRDXs) are a family of non-selenium-dependent glutathione peroxidases which destroy peroxides, organic hydroperoxides and peroxynitrite [23]. Glutaredoxins (GRX), also known as thioltransferases, are a family of glutathione-dependent thiol oxidoreductase enzymes important in the maintenance of thiol redox state; they catalyze the removal of GSH from proteins with a disulfide bridge; GRX2 is an isoform presents in mitochondria and nuclei [24]. Catalase (CAT) is primarily an intracellular enzyme that induces the decomposition of hydrogen peroxide to water and oxygen. Polynucleotide kinase/phosphatase (PNKP) is an enzyme involved in the repair of DNA strand breaks, including base excision repair, single and double-strand break repair. It possesses 3′-DNA phosphatase and 5′-DNA kinase activities useful to restore the strand structure and consequently to permit the action of DNA polymerases and ligases [25].

We aimed to test the hypothesis that hyperCHO might have an important role in the regulation of NLM by influencing consequently the transcription of genes for antioxidant proteins useful for the vitality, growth and aggressiveness of lymphoblastic lymphoma cells.

## Results

### How the high level of cholesterol influences lymphoblastic lymphoma cells

We have previously demonstrated that by enriching the culture medium with 800 nM CHO you reached a level of CHO corresponding to hyperCHO (280 mg/dl in the blood of patients). Therefore we used 800nM CHO to test the effect of hyperCHO on SUP-T1 cells.

**Fig. 1:**
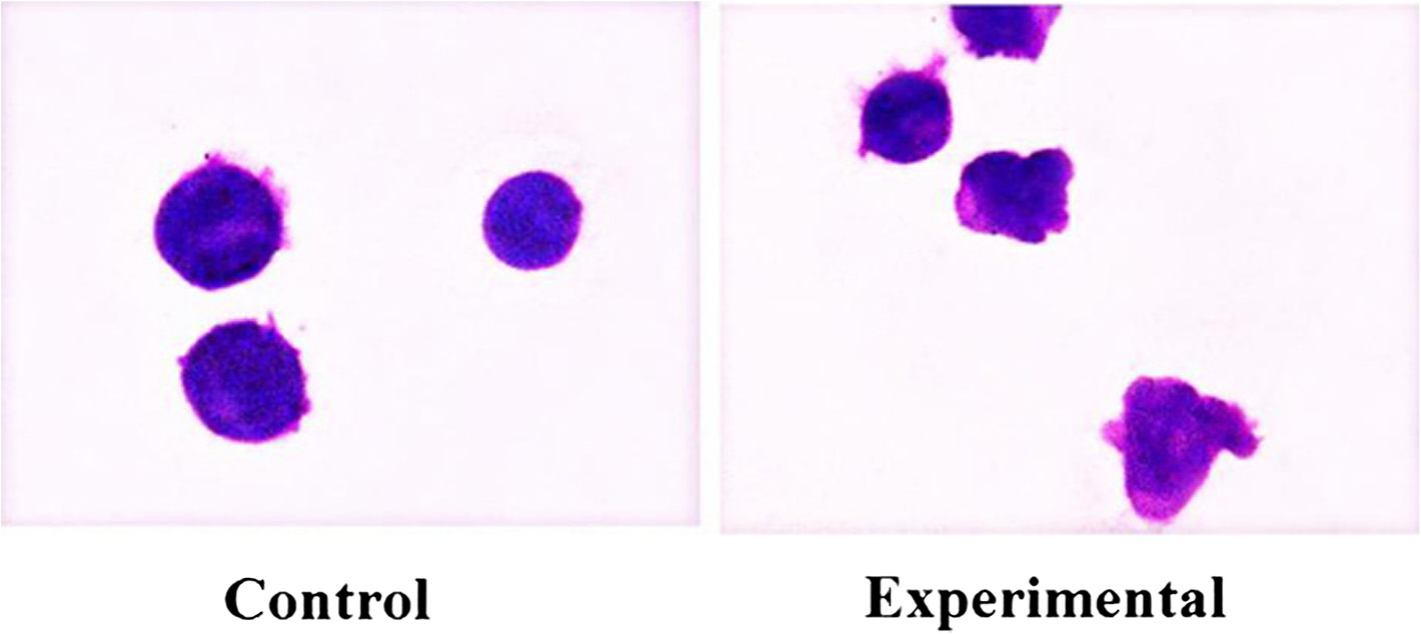
SUP-T1 morfology. The cells were cultured without (control) or with 800nM CHO (experimental) for three days, then they were treated as reported in Materials and Methods section

**Fig. 2:**
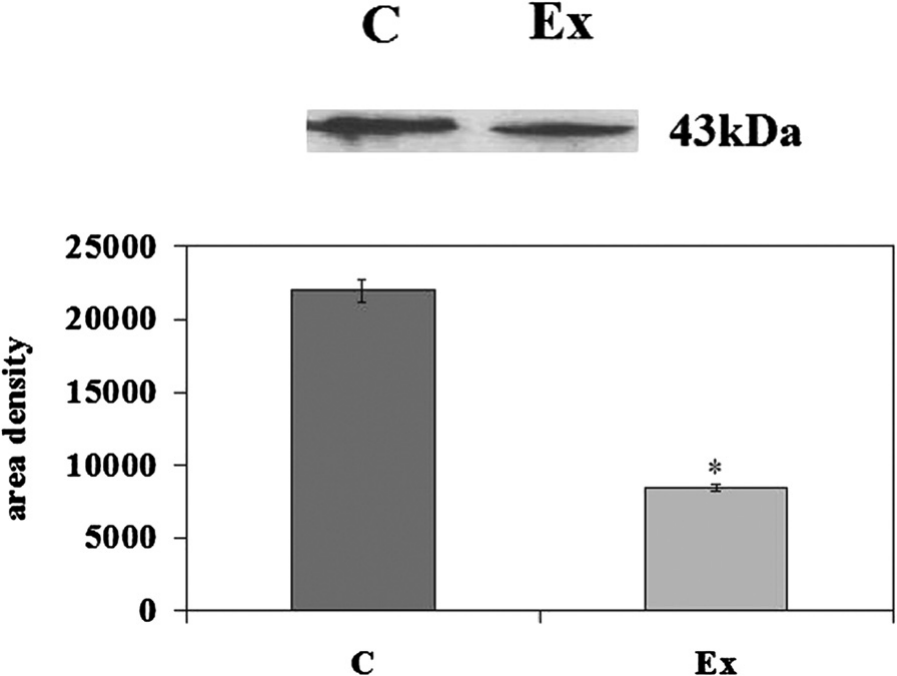
Effect of cholesterol on β-actin protein. Immunoblot of proteins was probed with anti-β-actin and visualized by ECL after 24 h of culture without (control) or with 800nM CHO (experimental). Apparent molecular weight (43 kDa) was calculated according to the migration of molecular size standards. The area density was calculated with Scion Image programme on densitometry scanning; the data represent the mean ± S.D. of three experiments performed in duplicate. (Significance, *P < 0.001 versus Control sample)

**Fig. 3:**
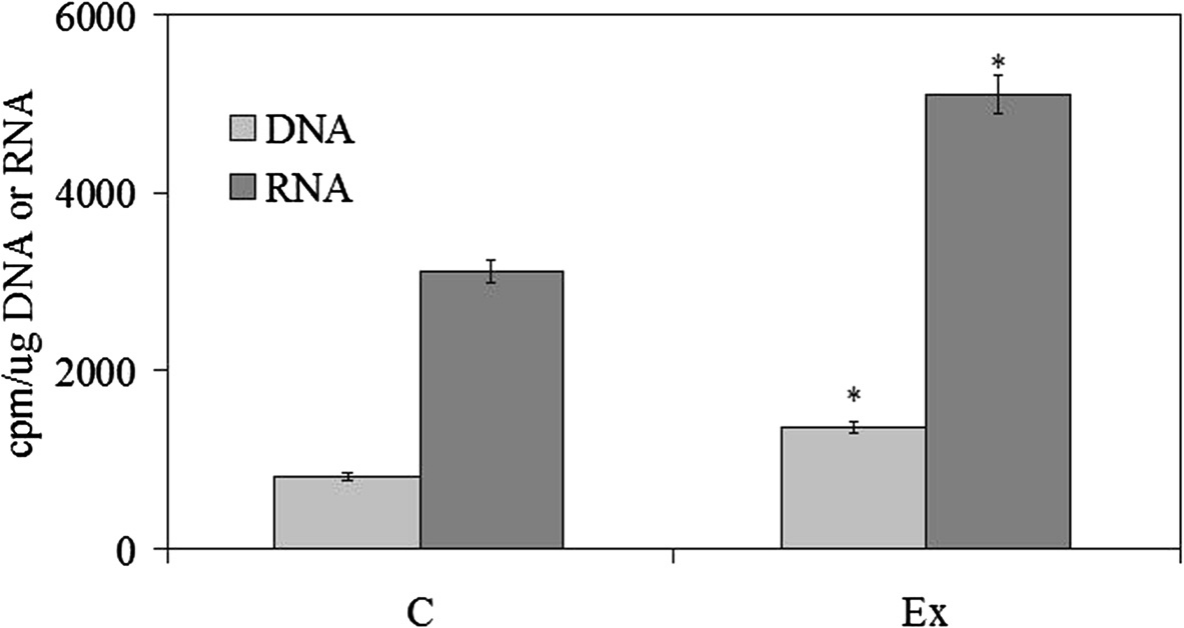
Effect of cholesterol on DNA and RNA synthesis. DNA synthesis was studied by evaluating the incorporation of ^3^H-thymidine in the DNA and RNA synthesis by evaluating the incorporation of ^3^H-UTP in the RNA. The specific activity was calculated as cpm/μg DNA and cpm/μg RNA, respectively. The data represent the mean ± S.D. of three experiments performed in duplicate. (Significance, *P < 0.001 versus Control sample)

**Fig. 4:**
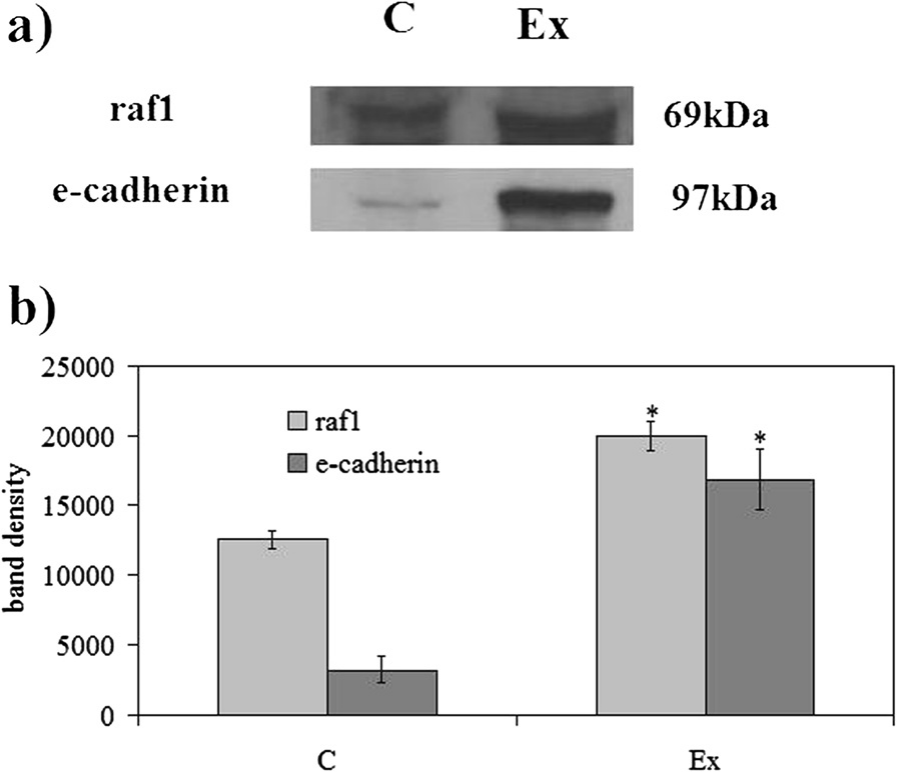
Effect of cholesterol on raf1 and e-cadherin proteins. **a** Immunoblot of proteins was probed with specific antibodies and visualized by ECL after 24 h of culture without (control) or with 800nM CHO (experimental). Apparent molecular weights were calculated according to the migration of molecular size standards; **b** The area density was calculated with Scion Image programme on densitometry scanning; the data represent the mean ± S.D. of three experiments performed in duplicate. (Significance, *P < 0.001 versus Control sample)

**Fig. 5:**
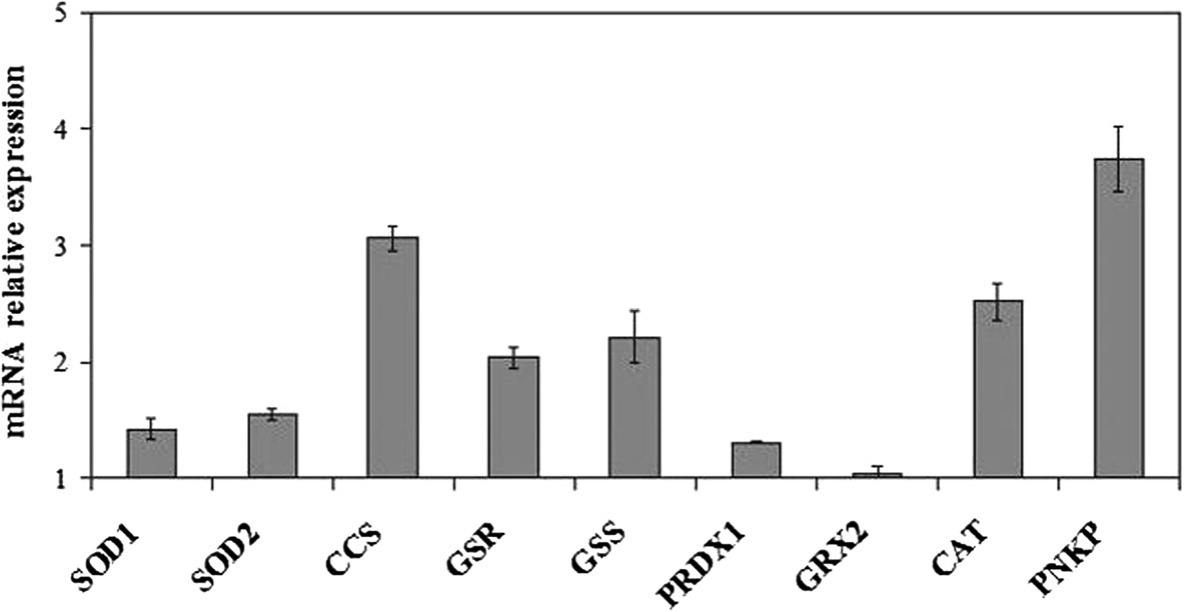
Effect of cholesterol on SOD1, SOD2, CCS, GSR, GSS, PRDX1, GRX2, CAT, PNKP expression. RTqPCR analysis was performed in control (without CHO) and experimental SUP-T1 cells (with 800nM CHO) collected after 24 h of culture. The results were normalized with the levels of the GAPDH and expressed as mRNA of experimental sample versus control sample. Data are expressed as the mean ± S.D. of 3 independent experiments performed in three PCR replicates

First we investigated the possible changes of cell morphology (Fig. 1). Hematoxylin-eosin staining of control cells showed round cells with very big nuclei intensely colored occupying almost the whole cell, as previously shown [26]. No significant differences were in the major and minor axis (4.37 μm ± 0.25); cell surface area was 19.96 ± 1.75 μm^2^. Experimental cells changed the shape, the major axis was 5.25 μm ± 1.07 and the minor axis was 3.91 μm ± 1.10; however cell surface area was similar to that of control sample (21.33 ± 1.30 μm^2^). Cellular protrusions were evident (Fig. 1). The expression fold of β-actin gene expression in Ex sample respect to C sample was 0.7888 ± 0.056, by indicating a gene down-regulation. The immunoblotting analysis of β-actin showed the presence of the band protein in C cells corresponding to 43 kDa apparent molecular weight (Fig. 2a); in Ex cells the band was reduced 2.62 times (Fig. 2b). To investigate if the high CHO levels can increase cancer cell malignancy, such as proliferation, migration and/or invasion, we analyzed first DNA and RNA synthesis after 48 h of cell culture. The results showed that 800nM CHO stimulated 1.68 times DNA synthesis and 1.63 times RNA synthesis (Fig. 3), by indicating that CHO influenced cell proliferation and activity. Then, we analyzed the behavior of raf1 and e-cadherin, as molecules involved in migration and/or invasion of cancer cell [27, 28]. The Fig. 4a showed that the band corresponding to 69 kDa apparent molecular weight, highlighted by anti-raf antibody, was increased after CHO treatment. The band area density analysis demonstrated that the value of raf1 was higher 1.59 times in Ex sample (Fig. 4b). High levels of CHO stimulated strongly e-cadherin (Fig. 4a) whose value increased 5.26 times as shown in Fig. 5, where the gene expression is referred to that of C cells. CHO treatment did not change GRX2 expression, overexpressed PRDX1 1.3 times, SOD1 and SOD2 about 1.5 times, GSR-GSS-CAT between 2.0 and 2.52 times, CCS 3.06 times and PNKP 3.75 times.

### HyperCHO influences nuclear lipid microdomains

**Fig. 6:**
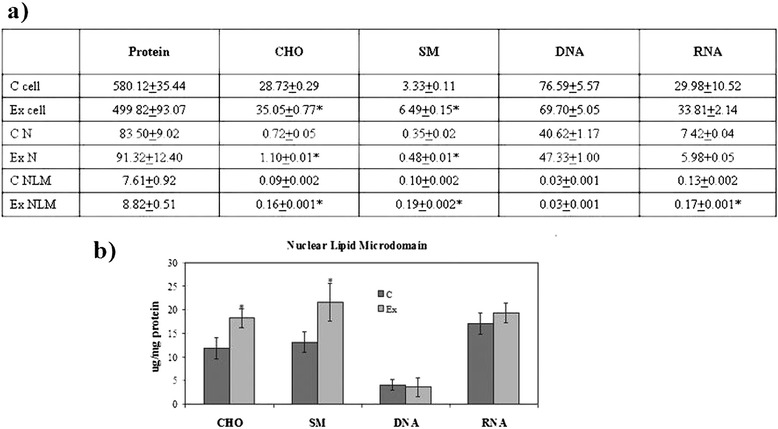
SUP-T1 composition. The cells were cultured without (control) or with 800nM CHO (experimental) and collected after 24 h. **a** cholesterol (CHO), sphingomyelin (SM), DNA and RNA content in cells, nuclei (N) and nuclear lipid microdomains (NLM), the data are expressed as μg/10^6^ cells and represent the mean ± S.D. of four experiments performed in duplicate; **b** CHO, SM, DNA and RNA content in NLM, the data are expressed as μg/mg protein and represent the mean ± S.D. of four experiments performed in duplicate. (Significance, *P < 0.001 versus Control sample)

**Fig. 7:**
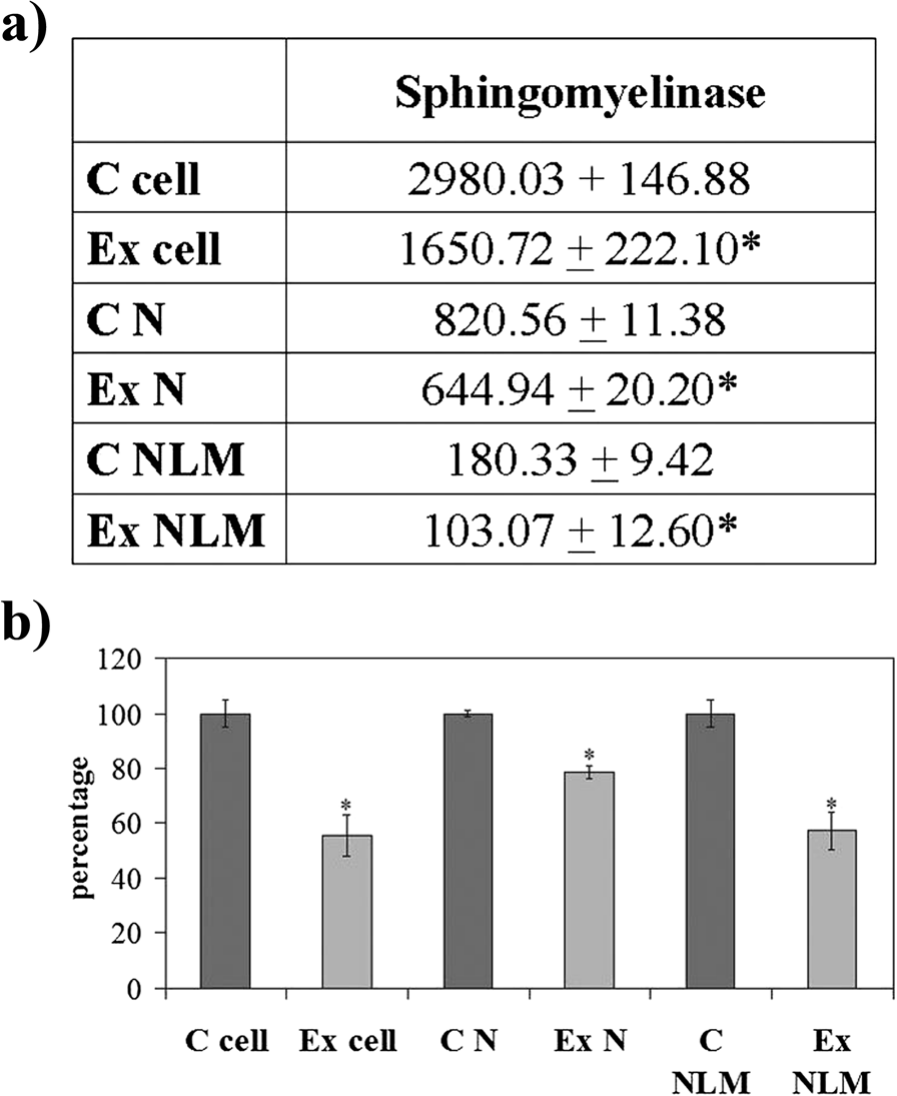
Sphingomyelinase activity. The cells were cultured without (control) or with 800nM CHO (experimental) and collected after 24 h; cell, nuclei (N) and nuclear lipid microdomains (NLM) were prepared as reported in Materials and Methods section. **a** The data are expressed as pmol/mg protein/min; **b** the data are expressed as percentage respect to control samples. (Significance, *P < 0.001 versus Control sample)

Since we previously demonstrated the role of the SUP-T1 NLM in transcription process [15], we tested the possibility that the culture medium hyperCHO could influence NLM composition and function. The level of N and NLM purification was similar to that previously reported [12, 15, 29]. In the Ex cells. the levels of protein and DNA expressed as μg/10^6^ cells did not change in cell, N and NLM in comparison with C samples; the level of RNA increased 1.31 times only in NLM. The level of CHO increased 1.59, 1.52, 1.78 in cell, N and NLM, respectively; the level of SM increased 1.94, 1.37, 1.90 in cell, N and NLM, respectively (Fig. 6a). If you express the data of NLM as μg/mg protein you can see that only CHO and SM changed (Fig. 6b). The SMase activity was inhibited 1.94 times in cells, 1.37 times in N and 1.90 times in NLM. (Fig. 7). By expressing the data as percentage of activity respect to the control samples, it was evident that the activity in cells and in NLM was more inhibited than in N (Fig. 7).

## Discussion

The main finding of this study is that by reproducing in vitro a condition of in vivo hyperCHO, lymphoma lymphoblastic cells overexpressed genes for antioxidant proteins (SOD1, SOD2, CCS, GSR, GSS and CAT) with the only exception of GRX2. These proteins counteract the oxidative stress that induces damage of lipids, nucleic acids and proteins, by altering their functions. It has been demonstrated that SOD2 polymorphism was associated with prostate cancer risk [30]. The sequestration of GSR by tumour cells yielded them less susceptible to oxidative stress, conferring a selective growth advantage in tumour cells [31]. The overexpression of GSS was in human colorectal cancer cell lines and tissues compared with normal samples [32]. PRDX1 was identified as a tumor-associated antigen in esophageal squamous cell carcinoma [33] and acted via mTOR/p70S6K pathway [34]; its overexpression was demonstrated in multiple myeloma [35], was associated with tumour angiogenesis and progression in human hepatocellular cancer [36], provided resistance to docetaxel treatment of lung cancer [37] and to alkylating agent bis-chloroethyl nitroso urea of oligodendroglial tumors [38]. Increased CAT levels were found in patients with non-small cell lung cancer compared to controls [39, 40].

We also showed that PNKP, as enzyme involved in the repair of DNA strand breaks was overexpressed after CHO incubation, by indicating that the cells were protected from the damage. On the other hand it was demonstrated that small inhibitors of PNKP increased the response to radiations of human lung carcinoma cells [41].

Therefore we reported the first observation in literature showing the CHO-antioxidant proteins- PNKP relationship in cancer. The results obtained might explain why the hyperCHO stimulated key proteins involved in cell cycle and survival (RNA polymerase II, STAT3, PKCzeta, PPARgamma and Cyclin D) and decreased Bax protein in SUP-T1 cells [6]. Here we showed that hyperCHO induced changes in cell shape with the appearance of protrusions that were notably associated with invasiveness and metastasis [40]. It was shown that tumour cell migration and invasion required actin cytoskeletal reorganization [42]; in addition, β-actin mutation was found in B-cell lymphoma [18]. Our results highlighted that the β-actin gene was downregulated and β-actin protein was reduced. The possibility that the β-actin was mutated also in our experimental model and therefore it was underestimated should be considered. In any case a change of β-actin might be responsible for the morphological modification of the cells. We have shown a correlation between hyperCHO and increase of DNA and RNA synthesis as well as raf1 and e-cadherin content, important molecules for cancer progression [27, 28]. The results obtained up to this point induced us to investigate about the possible mechanism of action of CHO in the regulation of gene expression. We previously demonstrated that CHO present in inner nuclear membrane linked SM to form NLM that acted as platform for active chromatin anchoring and gene expression [12, 13, 15]. So we wondered if the CHO, incorporated into the cells, might reach NLM by influencing their composition and, as a result, their function. Thus we demonstrated that hyperCHO is responsible for the enrichment of CHO in NLM and for the inhibition of SMase activity. In intranuclear environment existed two CHO-SM pools regulated by SMase, CHO-SM linked pool and CHO-SM free pool [43, 44]. The intranuclear CHO-SM linked pool was that present in NLM [12]. CHO, in turn, was able to regulate SMase [43, 44]. Thus it was possible to hypothesise that CHO inhibited SMase to have more SM to link and to enrich the nucleus of platforms for gene expression.

## Conclusion

In conclusion our data showed an association between hyperCHO and enrichment of NLM in inner nuclear membrane, increase of DNA and RNA synthesis and changes of antioxidant gene expression. It is possible to suggest that CHO, by keeping under control the oxidative status of cancer cells, might allow their vitality and growth.

## Methods

### Materials

Non-Hodgkin’s T cell human lymphoblastic lymphoma (SUP-T1) were obtained from Biological Materials Bank (ICLC)-CBA-Genoa. ^3^H palmytic acid, [^3^H] thymidine and [^3^H] UTP (41 Ci/mmol, 1.52 TBq/mmol) were obtained from Amersham Pharmacia Biotech (Rainham, Essex, UK); Ecoscint A was obtained from National Diagnostic (Atlanta, GA, USA); Thin layer chromatography (TLC) plates (silica Gel G60) were from Merck, Darmstadt, Germany; CHO, SM, Fetal bovine serum (FBS), RPMI 1640 Medium, PSF (penicillin, streptomycin and fungizone) were purchased from Sigma Aldrich Co. (St. Louis, MO, USA). Anti-actin, anti-Raf1, anti-phosphoRaf1 and anti-E-cadherin antibodies were obtained from Santa Cruz Biotechnology, Inc. (California, USA).

### Cell culture and treatments

SUP-T1 cells were grown as previously reported [6]. For each experiments, two lots of cells were prepared: the control sample (C) without CHO and the experimental sample (Ex) with 800nM CHO. After 24 h of culture, part of the cells were used for their analysis and part for Nuclei (N) and Nuclear Membrane Microdomains (NLM) purification.

### Cell morphology

SUP-T1 cells, cultured for three days, were fixed and submitted to the hematoxylin-eosin staining as previously reported [26] and analyzed by inverted microscopy EUROMEX FE 2935 (ED Amhem, The Netherland) equipped with a CMEX 5000 camera system (40x magnification). The analysis of cell size was performed by ImageFocus software.

### DNA and RNA synthesis

The DNA and RNA synthesis was studied in synchronized cells by using [^3^H]-thymidine or [^3^H]-UTP as precursor, previously reported [44, 45]. After syncronization, the cells were cultured for 48 h of in the absence (C) or presence (Ex) of 800nM CHO; 1 μCi [^3^H]-thymidine or 1 μCi [^3^H]-UTP was added to the culture medium 3 h before the analysis. The data of the radioactivity were referred to the DNA or RNA amount and expressed as cpm/μg DNA and cpm/μg RNA, respectively.

### Nuclei purification

The nuclei (N) were purified from 20x10^6^ SUP-T1 cells and checked for possible cytoplasmic contamination as previously reported [43].

### Nuclear Lipid Microdoman purification

The Nuclear Lipid Microdomains (NLMs) were purified from 1x10^9^ SUP-T1 cells and checked for possible and nuclear contamination as previously reported [15].

### Biochemical determinations

Protein, DNA and RNA contents were determined as previously reported [43].

### Western blotting

About 30 μg of cell proteins were submitted to SDS-PAGE electrophoresis in 10 % polyacrylamide slab gel. Proteins were transferred into nitrocellulose for 90 min as previously described [12]. The membranes were blocked for 30 min with 0.5 % no-fat dry milk in PBS (pH 7.5) and incubated overnight at 4 °C with the β-actin specific antibody. The blots were treated with HRP-conjugated secondary antibodies for 90 min. Visualization was performed with the enhanced chemiluminescence kit from Amersham (Rainham, Essex, UK). The apparent molecular weight of β-actin, raf1 and e-cadherin was calculated according to the migration of molecular size standards. The area density of the bands was evaluated by densitometry scanning and analyzed with Scion Image.

### Lipid analysis

Lipids were extracted and CHO measured, after thin-layer chromatography (TLC) separation, as previously reported [46]. To evaluate SM the cells were incubated with 1 μCi/ml of ^3^[H] palmitic acid, diluted with cold palmitic acid as previously reported [15]. After 24 h, the cells from 5 flasks were pooled and NLM were purified. The lipids were extracted, separated on TLC and analysed as reported by Cascianelli et al. [6]. The radioactivity was measures in counting vials with 10 ml Ecoscint A and 1 ml water and the radioactivity was measured with a Packard liquid scintillation analyser (Packard Instrument Company, Meriden, CT, USA).

### Sphingomyelinase activity

The sphingomyelinase (SMase) activity was detected according to Albi et al. [47]. Incubations were performed at 37 °C for 45 min. The reaction was stopped by adding 2 ml chloroform and methanol (2:1), 0.4 ml of 0.5 % NaCl was added to the tubes and vortexed. After centrifugation at 2000 RPM × 10 min, the upper phase was removed and 0.5 ml was diluted in counting vials with 10 ml Ecoscint A and 1 ml distilled water; radioactivity was measured with a Packard liquid scintillation analyzer.

### Reverse transcription quantitative PCR (RTqPCR)

Control and experimental SUP-T1 cells were used for total RNA extraction performed by using RNAqueous ®-4PCR kit (Ambion Inc., Austin, Texas), as previously reported [25]. cDNA was synthesized under the following conditions: 50 °C for 2 min, 95 °C for 10 min, 95 °C for 15 s and 60 °C for 1 min for 40 cycles. RTqPCR was performed using Master Mix TaqMan®Gene Expression and 7.300 RT-PCR instrument (Applied Biosystems), targeting β-actin (ACTB; Hs99999903), Superoxide dismutase 1 (SOD1; Hs00533490_m1), Superoxide dismutase 2 (SOD2; Hs00167309_m1), Copper Chaperone for SOD (CCS; Hs00192851_m1), Glutathione reductase (GSR; Hs00167317_m1), Glutathione synthase (GSS; Hs00609286_m1**),** Peroxiredoxins (PRDXs; Hs00602020_mH), Glutaredoxin2 (GRX2; Hs00375015_m1**),** Catalase (CAT; Hs00156308_m1), Polynucleotide kinase/phosphatase (PNKP; Hs00892544_m1). The results were normalized with the levels of the Glyceraldehyde 3-phosphate dehydrogenase (GAPDH; Hs02758991_g1) housekeeping gene.

### Statistical analysis

Three experiments in duplicate where performed for each analysis. The data are expressed as mean ± S.D. and *t* test was used for statistical analysis.

## Abbreviations

CAT: catalase
CCS: copper chaperone for SOD
CHO: cholesterol
GRX: glutaredoxins
GSH: glutathione
GSR: GSH reductase
GSS: glutathione synthase
LMs: lipid microdomains
NLMs: nuclear lipid microdomains
PNKP: polynucleotide kinase/phosphatase
PRDXs: peroxiredoxins
SODs: superoxide dismutases
SM: sphingomyelin.
